# Factors associated with poor sleep quality among dental students in Malaysia

**DOI:** 10.7717/peerj.17522

**Published:** 2024-06-27

**Authors:** Khor Yong Jie, Noraini Mohamad, Munirah Mohd Adnan, Nor Azlida Mohd Nor, Nor Faharina Abdul Hamid, Zurainie Abllah

**Affiliations:** 1 School of Dental Sciences, Health Campus, Universiti Sains Malaysia, Kubang Kerian, Kelantan, Malaysia; 2 Department of Community Oral Health & Clinical Prevention, Faculty of Dentistry, Universiti Malaya, Wilayah Persekutuan, Kuala Lumpur, Malaysia; 3 Faculty of Dentistry, Universiti Teknologi MARA, Sungai Buloh Campus, Jalan Hospital, Sungai Buloh, Selangor, Malaysia; 4 Department of Paediatric Dentistry and Dental Public Health, Kulliyyah of Dentistry, IIUM Kuantan Campus, Kuantan, Pahang

**Keywords:** Academic performance, Dental students, Sleep quality, Skip class

## Abstract

**Background:**

Good sleep quality is crucial for dental students as they must have optimal cognitive function, memory, and decision-making to accomplish their learning requirements. This study aims to determine sleep quality, its associated factors, and the association between sleep quality and academic performance among dental students in Malaysia.

**Methods:**

This cross-sectional study involved dental students at four public universities in Malaysia. A validated Pittsburgh Sleep Quality Index (PSQI) questionnaire was used to assess their sleep quality. An additional self-administered questionnaire was employed to obtain the students’ sociodemographic profile, lifestyle, and academic performance. The data were analysed using descriptive, chi-square, and multiple logistic regression.

**Results:**

Three hundred eighty-four dental students participated in this study. About half of the dental students (51.6%) have poor sleep quality. The mean of sleep hours per night was 5.72 (SD 1.06). The sleep quality was significantly poor among Malay students (*P* = 0.023), students who stayed at hostel (*P* = 0.002), and those who consumed caffeinated drinks (*P* = 0.028). Multiple logistic regression analysis revealed that the poor sleep quality was significantly associated with self-perceived poor academic performance (Adjusted Odds Ratio (AOR) 2.95, 95% CI [1.25–6.96], *P*-value = 0.013) and students skipping class (AOR 1.70, 95% CI [1.00–2.91], *P*-value = 0.046).

**Conclusions:**

Most of the dental students in Malaysia have poor sleep quality. Ethnicity, accommodation, and caffeine consumption were significantly associated with sleep quality. Awareness to sleep quality among dental students is needed to ensure they are able to cope with the challenging dental school learning environment.

## Introduction

Sleep is crucial for maintaining good health and well-being at all stages of life. Adequate high-quality sleep is essential for physiological repair and recovery. However, its insufficiency has been recognized as an increasing public health issue. Adverse sleep problems can heighten the likelihood of health issues, while certain diseases and disorders can also impact the quantity and quality of sleep in individuals (Seow et al., 2020). Sleep quality encompasses the quantitative aspects of sleep such as sleep quantity, sleep latency, or number of arousals at night, as well as subjective factors such as sleep depth, feeling of restfulness upon waking, and general satisfaction with sleep (Pilcher, Ginter & Sadowsky, 1997).

Sleep duration and quality have several effects on human health. Previous research has shown that poor sleep quality and short sleep duration were associated with a higher prevalence of depressive symptoms and may become risk factors for mental health disorders among university students (Li et al., 2020). Further, sleep among university students varies considerably across different cultural populations and regions. It was found that Japanese university students sleep less than Canadian university students. However, despite the less sleep compared to that of the Canadian university students, Japanese university students reported being less tired and having better health, indicating that cultural differences emerged as significant parameters of sleep (*e.g*. sleep time) and beliefs about sleep (*e.g*. perceived relation between sleep and health) (Cheung et al., 2021).

Sleep plays a vital function in enhancing cognitive abilities, particularly memory retention. Poor nighttime sleep quality and daytime lethargy negatively affect students’ physical and cognitive health and academic performance (Maheshwari & Shaukat, 2019). Sleep disorders are among the most common health problems for late adolescents and young adults. Students usually report lack of sleep, or difficulty in sleeping (Becker et al., 2018). Studies have shown that, poor sleep quality among university or college students in Asia was high ranging from 50–58% (Cheng et al., 2012; Ji & Wang, 2018; Suen, Tam & Hon, 2010). In Malaysia, research has revealed that 60% of health sciences students and 59.6% of medical students had poor sleep quality (Saat et al., 2020; Said et al., 2020). The percentage was even higher among dental students. This is demonstrated by studies conducted in Saudi Arabia and Brazil which found that 72.5% and 65.2% of the dental students had poor sleep quality respectively (Elagra et al., 2016; Muñoz et al., 2023). This could be related to dental specialty which often comprises heavy didactic and clinical contents, causing a lot stress on students and requiring extensive study and long practical sessions (Elagra et al., 2016). For students to succeed and master their learning requirements, a restful night’s sleep is crucial for optimal cognitive function, memory, and decision-making. Additionally, getting enough rest will give them the energy, stamina, and strength they need to complete the learning program (Said et al., 2020).

Several factors have been identified by researchers as being associated with poor sleep quality. A study involving university students in Taiwan found that, poor sleep quality was significantly associated with undergraduate students, female gender, the habit of skipping breakfast, tea drinking, a higher tendency toward internet addiction, poor social support and higher neuroticism (Cheng et al., 2012). A study among medical students in Malaysia found that students in the clinical year were less likely to have poor sleep quality than pre-clinical students. Nonetheless, students with depression symptoms, compared to those who did not have depression have a 1.71 times higher risk of having poor sleep quality (Said et al., 2020). Another study involving medical students in Saudi Arabia reported that students who do not suffer from stress are less likely to have poor sleep quality, whereas the risk of having poor sleep quality is almost four times higher in students with cumulative grade point average (GPA) less than 4.25 (Almojali et al., 2017). The previous studies mainly focus on undergraduate students taking different programs, and there is a lack of data on how sleep quality affects dental students and their academic performance. Therefore, this study is conducted to assess the factors associated with poor sleep quality among dental students at Malaysian universities.

Poor sleep quality might affect students learning progress which leads to poor academic performance and their performance as a healthcare professional in the future (Haque et al., 2018). Inadequate duration and poor quality of sleep also negatively affect their concentration and cognitive function (Hanapi et al., 2021;Suen, Tam & Hon, 2010). Sleep deprivations are commonly related to daytime sleepiness and declined level of attention which affects performance (Siraj et al., 2014). A study among dental students in Saudi Arabia found that, poor sleep quality was associated with lower academic performance, especially in clinical years (Elagra et al., 2016). In Malaysia, a study among medical students found that respondents who slept less than 6 h during the weekend had significantly lower cumulative GPA compared to those with average sleep between 6 to 8 h and those who sleep more than 8 h (Siraj et al., 2014). A study also found that sleep frequent sleep disruptions negatively impact students’ academic performance, creating a vicious cycle (Abdulghani et al., 2012). Additionally, poor sleep also affects individual performance by increasing depression, decreasing motivation, and compromising health (Kazim & Abrar, 2011). However, whether similar factors influence dental students’ sleep quality and their academic performance remains unexplored. Therefore, this study is conducted to assess sleep quality and its associated factors among Malaysian university dental students. In addition, the association between sleep quality and academic performance of Malaysian university dental students is assessed. Understanding the factors that affect sleep quality among dental students’ and how it affects their academic achievement could help to develop targeted interventions to enhance their overall performance.

## Methods

### Population and sample

This cross-sectional study involved dental students from Universiti Sains Malaysia, Universiti Malaya, International Islamic University Malaysia, and Universiti Teknologi MARA. It was conducted from 15^th^ July 2023 to 30^th^ August 2023. Dental students from 2 to Year 5 who consented to participate were included in this study. First-year dental students were excluded because during data collection, they had not yet sat for the final exam. The convenience sampling method was applied in this study. The sample size was calculated using the single proportion formula at 95% confidence interval (CI) based on the objective to determine the proportion of sleep quality among the dental students. The expected proportion was estimated at 65%, which was the percentage described by dental students in Saudi Arabia as good or very good sleep quality (Elagra et al., 2016). Sample sizes were calculated for various precisions, and a sample size of 348 was chosen with a precision of 0.05, considering the available resources. This calculation ensured sufficient test power to detect meaningful differences in sleep quality within the sampled population. Anticipating a 10% non-response rate, the final sample size was 384 for this study.

### Ethical consideration

This study was approved by the Human Research and Ethics Committee, Universiti Sains Malaysia (USM/JEPeM/22040222) on 5 June 2022, Medical Ethics Committee, Faculty of Dentistry, University of Malaya (DF CO2204/0051(L)) on 27 June 2022, International Islamic University Malaysia (IIUM) Research Ethics Committee (IREC 2022-045) on 27 April 2022 and Universiti Teknologi Mara (UiTM) Research Ethics Committee on (REC/07/2022 (ST/MR/131) on 4 July 2022.

### Research tools

An online questionnaire using Google Forms was used to collect variables of interest in this study. The questionnaire consisted of four parts. The first part included questions on the participants’ demographic characteristics, including age (years), gender (male/female), ethnicity (Malay/Chinese/Indian/Others), parents’ monthly income (in MYR), marital status (single/married), body mass index (BMI), current academic years, place of study, accommodation (hostel/rental/family house), and presence of any medical problems (yes/no). For BMI, it was recategorized as underweight (BMI < 18.5 kg/m^2^), normal (BMI 18.5–22.9 kg/m^2^), overweight (BMI 23–27.4 kg/m^2^) and obese (≥27.5 kg/m^2^) (Ministry of Health Malaysia, 2023).

The second part of the questionnaire focused on activities or habits related to sleep quality. The first item was on exercise for at least 30 min for each session for the past one month (yes/no). If respondents answered “yes”, they were asked to specify the frequency of their exercise over the past months as either >5 times per week, 3–5 times per week, or <3 times per week. This frequency of exercise was determined based on recommendations for healthy adults regardless of age, whereby the duration of exercise should be at least 150 min/week for moderate intensity exercise (Samitz, Egger & Zwahlen, 2011). Other items include eating sleeping pills to improve sleep for the past 1-month, electronic device uses before bedtime for the past 1 month, smoking for the past 1 month, and alcohol and caffeinated drink consumption for the past 1 month. The respondents answered “yes” or “no” for these items (Pham et al., 2021). If the respondents answer “yes” for consumption of caffeinated drink, they need to choose either they rarely consume a caffeinated drink, 1 day per week, 2–3 days per week, 4–6 days per week or every day. These response option were adapted from a study conducted by Pham et al. (2021).

The third part of the questionnaire was on the students’ performance in the class. The questionnaire includes 1) self-perceived academic performance in the current academic year including very good, good, average, poor and very poor 2) falling asleep during class 3) skipping class, 4) coming late to the class and 5) involvement in extracurricular activities. The fourth part of the questionnaire assessed the students’ sleep quality using the PSQI questionnaire. The questionnaire was developed and validated by Buysse et al. (1989) in 1989 and was used with permission from the authors. The questionnaire has good internal consistency, with a Cronbach’s alpha of 0.83 (Buysse et al., 1989). The PSQI was divided into 10 questions which comprise 19 items forming seven components: (1) sleep quality (one item), (2) sleep latency (two items), (3) sleep duration (one item), (4) sleep efficiency (three items), (5) sleep disturbance (nine items), (6) sleep medication (one item), and (7) daily dysfunction (two items). The seven component scores were then summed to yield a global PSQI score ranging from 0–21, with higher scores indicating worse sleep quality and a cut-off score above five indicating poor sleep quality (Buysse et al., 1989).

### Data collection

Data collection was conducted online *via* a self-administered questionnaire using Google Forms link from 7 July 2022 to 31 August 2022. The link to the questionnaire, the information sheet, and consent form were distributed using WhatsApp to all participants through each university representative. The first section of the questionnaire contained an explanation of the survey’s purpose and confidentiality. On the first page, the participants must indicate their agreement and consent to participate in the study. The consent form was designed to be skipped. If participants selected “No” in response to the consent query, they were redirected to a page, thanked for their time, and exited the form. If they selected “Yes,” indicating their agreement to participate, they were redirected to answer the questionnaires.

### Statistical analysis

Data entry and analysis were carried out using IBM SPSS Statistics for Windows (version 27.0, IBM Corp., Armonk, NY, USA). Data checking and cleaning were performed before the analysis. Descriptive analysis was used to describe the sociodemographic characteristics of the respondents and to determine the proportion of good and poor sleep quality among dental students of Malaysian universities. Numerical data were presented as a mean and standard deviation (SD), and categorical data were presented as frequency and percentage. A Chi-square and Fisher’s exact test were used to determine the factors associated with sleep quality among the dental students. Simple and multiple logistic regression analyses (MLR) were used to determine the association between poor sleep quality and academic performance of Malaysian university dental students. The independent variables included self-perceived academic performance, falling asleep in class, skipping class, coming late to class and involvement in extracurricular activities. The dependent variable was the total score of PSQI items with score above five indicating poor sleep quality and score less than or equal to five indicating good sleep quality. Before MLR was performed, the distribution and frequencies were examined. Simple logistic regression (SLR) analysis was done to screen variables for subsequent analysis using multiple logistic regression (MLR). All variables with a *P*-value less than 0.25, as well as clinically relevant variables identified from the SLR, were included in the multivariable analysis (multiple logistic regression). The *P*-value was set larger than the conventional level of significance to enable the inclusion of more clinically relevant variables from the SLR analysis into the model of MLR analysis (Zhang, 2016). The interaction terms were checked using the likelihood ratio test. Multicollinearity-related issues were identified by the variance inflation factor test. The final model was assessed for fitness using the Hosmer-Lemeshow goodness-of-fit test. The sensitivity and specificity, classification table and the area under the receiver operating characteristic (ROC) curve were also obtained to evaluate the model fitness. A *P*-value less than 0.05 was considered as statistically significant.

## Results

The target sample size of this study was 384 respondents. The Google Forms link was deactivated once this number was reached, ensuring the desired sample size was achieved. None of the participants were excluded, and all who accessed the survey provided complete responses, resulting in a 100% completion rate. The respondents’ sociodemographic profile and BMI status are shown in Table 1. The mean age of respondents was 22.63 ± 1.35 years and it ranged between 20 to 26 years old. The majority were female (79.7%), single (97.9%) and Malay (82.0%). The median parents’ income per month was MYR7000 (IQR7238) or USD 1,487. About half of the respondents had a normal BMI (49%) and most lived in a hostel (90%).

**Table 1:** Sociodemographic data and BMI status of the respondents (*n* = 384).

Variables	Frequency (%)	Mean (SD)
Age		22.63 (1.35)^a^
Gender		
Male	78 (20.3%)	
Female	306 (79.7%)	
Ethnicity		
Malay	315 (82.0%)	
Chinese	45 (11.7%)	
Indian	11 (2.9%)	
Others	13 (3.4%)	
Parents’ income (MYR)	7,000^b^ (7,238)^c^	
Marital status		
Single	376 (97.9%)	
Married	7 (2.1%)	
Body mass index (BMI)		
Underweight (BMI < 18.5 kg/m^2^)	63 (16.4%)	22.02 (4.00)
Normal (BMI 18.5–22.9 kg/m^2^)	188 (49.0%)	
Overweight (BMI 23–27.4 kg/m^2^)	95 (24.7%)	
Obese (≥27.5 kg/m^2^)	38 (9.9%)	
Current academic years		
Second year	108 (28.1)	
Third year	100 (26.0)	
Fourth year	75 (19.5)	
Fifth year	101 (26.4)	
Place of study		
Universiti Sains Malaysia (USM)	120 (31.3%)	
Universiti Malaya (UM)	43 (11.2%)	
International Islamic University Malaysia (IIUM)	103 (26.8%)	
Universiti Teknologi MARA (UiTM)	118 (30.7%)	
Accommodation		
Hostel	372 (96.9%)	
Rental/family house	12 (3.1%)	
Medical problems		
Yes	30 (7.8%)	
No	354 (92.2%)	

**Notes:**

a Range of age, minimum: 20, maximum: 26

b MYR 7,000 = USD 1,487

c Median (IQR)

The respondents’ lifestyle that might affect sleep quality is shown in Table 2. About two-thirds (72.1%) of the respondents performed exercise and more than half (62.1%) exercised less than three times per week. Most respondents (96.4%) are non-smokers and use electronic devices before sleep (92.5%). Only 4.9% of the respondents’ drank alcohol. Most of the respondents (78.9%) consumed a caffeinated drink. The average cup of caffeinated beverage consumed per week was 2.77 ± 3.11 cups.

**Table 2:** Respondents’ lifestyle (*n* = 384).

Variables	Frequency (%)
Exercise for the past 1 month	
Yes	277 (72.1%)
No	107 (27.9%)
Frequency of exercise (*n* = 277)	
>5 times per week	28 (10.1%)
3–5 times per week	77 (27.8%)
<3 times per week	172 (62.1%)
Eating sleeping pills for the past 1 month	
Yes	4 (1.0%)
No	380 (99.0%)
Electronic device used before sleep for the past 1 month	
Yes	355 (92.5%)
No	29 (7.5%)
Frequency of electronic devices used before sleep (*n* = 355)	
>5 times per week	317 (89.3%)
3–5 times per week	30 (8.5%)
<3 times per week	8 (2.2%)
Smoking for the past 1 month	
Yes	14 (3.6%)
No	370 (96.4%)
Alcohol consumption for the past 1 month	
Yes	19 (4.9%)
No	365 (95.1%)
Caffeinated drink consumption for the past 1 month	
Yes	303 (78.9%)
No	81 (21.1%)
Frequency of caffeinated drink consumption (*n* = 303)	
Rarely consume a caffeinated drink	68 (22.4%)
1 day per week	35 (11.6%)
2–3 days per week	93 (30.7%)
4–6 days per week	53 (17.5%)
Everyday	54 (17.8%)

The respondents’ academic performance is shown in Table 3. More than half (68.5%) rated themselves as average in self-perceived academic performance, while about one-third of the respondents sometimes (37.0%) fell asleep in class. The majority did not skip class (82.0%) and did not come late to class (74.0%). About one-third (31.8%) were sometimes involved in extracurricular activities.

**Table 3:** Distribution of respondents by academic performance (*n* = 384).

Variables	Frequency (%)
Self-perceived academic performance	
Very good	5 (1.3%)
Good	84 (21.9%)
Average	263 (68.5%)
Poor	32 (8.3%)
Very poor	0 (0%)
Fallen asleep in class	
Never	78 (20.3%)
Seldom	101 (26.3%)
Sometimes	142 (37.0%)
Often	47 (12.2%)
Very often	16 (4.2%)
Skip class	
Yes	69 (18.0%)
No	315 (82.0%)
Frequency of skip class (*n* = 69)	
<3 times per month due to illness	26 (37.7%)
three times or more per month due to illness	4 (5.8%)
<3 times per month due to other reason	34 (49.3%)
three times or more per month due to other reason	5 (7.2%)
Come late to the class	
Yes	100 (26.0%)
No	284 (74.0%)
Frequency of come late to class (*n* = 100)	
<3 times per month due to illness	39 (39.0%)
three times or more per month due to illness	12 (12.0%)
<3 times per month due to other reason	43 (43.0%)
three times or more per month due to other reason	6 (6.0%)
Involvement in extracurricular activities	
Never	101 (26.3%)
Seldom	98 (25.5%)
Sometimes	122 (31.8%)
Often	41 (10.7%)
Very often	22 (5.7%)

**Figure 1:**
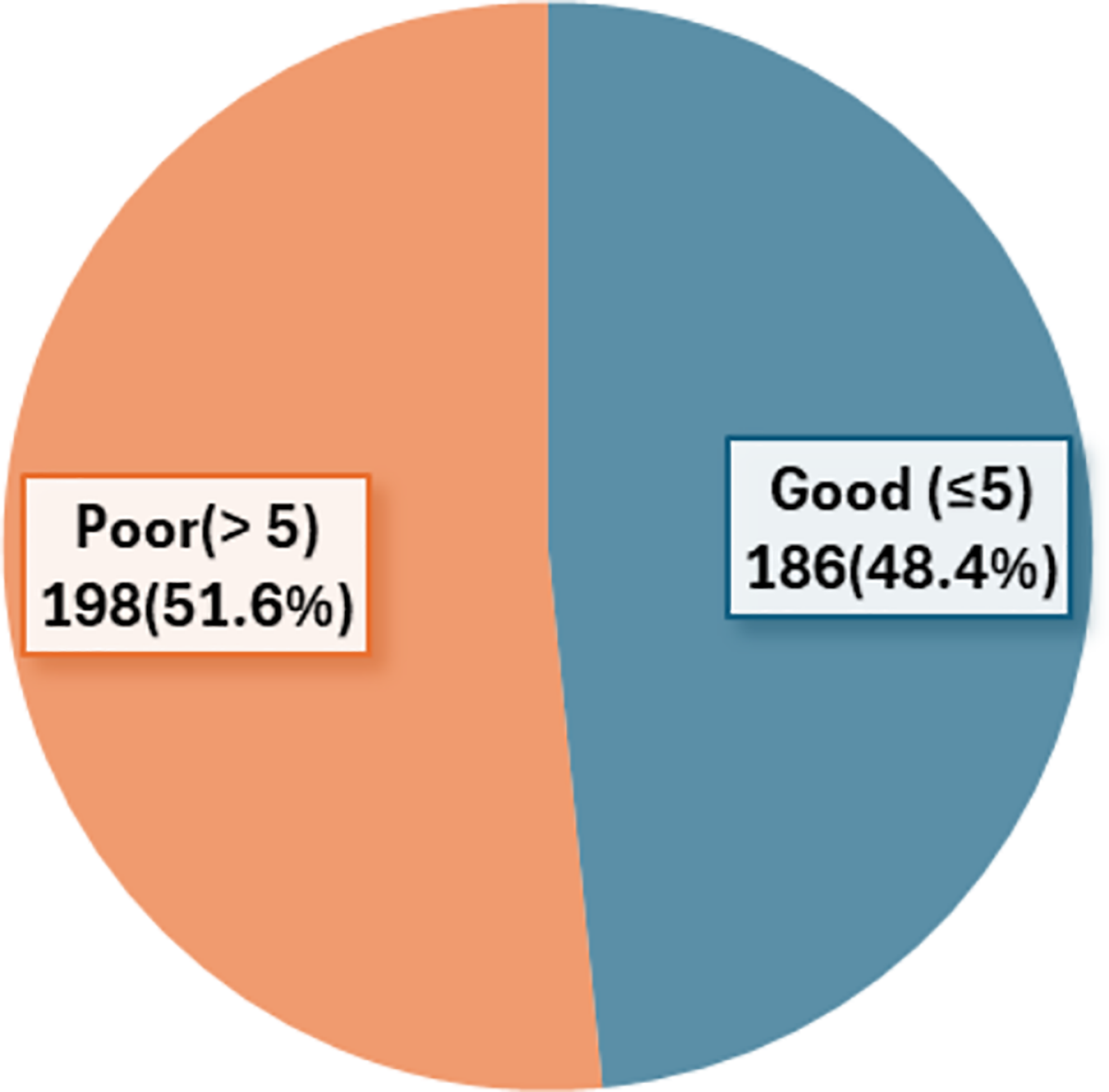
Percentage of total PSQI global score category.

Table 4 shows the distribution of respondents by sleep quality elements. The average sleep duration of the respondents was 5.72 ± 1.06 h. The time taken to fall asleep at night ranged between 1 and 120 min with 43.8% of the respondents having sleep latency between 16 and 30 min. Meanwhile, the habitual sleep efficiency domain shows that the average sleep efficiency is 94.50 ± 9.65. Figure 1 shows that about half (51.6%) of the respondents had poor sleep quality.

**Table 4:** Distribution of respondents by sleep quality elements (*n* = 384).

Element of sleep quality	Frequency (%)	Mean (SD)
Sleep latency (min)		
Less than 15	127 (33.1%)	16.09 (16.34)
16–30	168 (43.8%)	
31–60	72 (18.8%)	
More than 60	17 (4.3%)	
Sleep duration (h)		
>7	71 (18.5%)	5.72 (1.06)
6–7	156 (40.6%)	
5–6	152 (39.6%)	
<5	5 (1.3%)	
Habitual sleep efficiency (%)		
>85	322 (83.9%)	94.50 (9.65)
75–85	41 (10.7%)	
65–75	17 (4.4%)	
<65	4 (1.0%)	
Sleep disturbances		
None	38 (9.9%)	
Mild	288 (75.0%)	
Moderate	57 (14.8%)	
Severe	1 (0.3%)	
Use of sleeping medication		
None	371 (96.7%)	
Mild	7 (1.8%)	
Moderate	4 (1.0%)	
Severe	2 (0.5%)	
Daytime dysfunction		
None	48 (12.5%)	
Mild	245 (63.8%)	
Moderate	86 (22.4%)	
Severe	5 (1.3%)	

Table 5 shows the factors associated with poor sleep quality among the dental students. The sleep quality of the dental students was significantly poor among Malay ethnicity students who stayed at hostel and consumed caffeinated drinks.

**Table 5:** Factors associated with sleep quality among Malaysian University dental students (*n* = 384).

Variables	Sleep quality *n* (%)		χ^2^ (df)	*P* value
	Poor	Good		
Age				
20–22	101 (26.3)	93 (24.2)	0.39 (1)	^a^0.843
23–26	97 (25.3)	93 (24.2)		
Academic years				
Second year-third year	108 (28.1)	100 (26.0)	0.24 (1)	^a^0.878
Fourth year-fifth year	90 (23.4)	86 (22.5)		
Gender				
Male	39 (10.2)	39 (10.2)	0.96 (1)	^a^0.757
Female	159 (41.4)	147 (38.2)		
Ethnicity				
Malay	171 (44.5)	144 (37.5)	5.205 (1)	^a^0.023
Others	27 (7.1)	42 (10.9)		
Marital status				
Single	196 (51.0)	182 (47.5)		^b^0.314
Married	2 (0.5)	4 (1.0)		
Body mass index (BMI)				
Underweight (BMI < 18.5 kg/m^2^)	35 (9.1)	28 (7.3)	3.345 (2)	^a^0.188
Normal (BMI 18.5–22.9 kg/m^2^)	88 (22.9)	100 (26.0)		
Overweight (BMI 23–27.4 kg/m^2^)/Obese (≥27.5 kg/m^2^)	75 (19.5)	58 (15.2)		
Accommodation				
Hostel	197 (51.3)	175 (45.6)	9.268 (0.1)	^a^0.002
Rental/ Family house	1 (0.3)	11 (2.8)		
Medical problems				
Yes	20 (5.2)	10 (2.6)	2.973 (1)	^a^ 0.085
No	178 (46.4)	176 (45.8)		
Exercise for the past 1 month				
Yes	142 (37.0)	135 (35.2)	0.036 (1)	^a^0.85
No	56 (14.6)	51 (13.2)		
Eating of sleeping pills for the past 1 month				
Yes	3 (0.8)	1 (0.3)		^b^0.624
No	195 (50.8)	185 (48.1)		
Electronic device use before sleep for the past 1 month				
Yes	185 (48.2)	170 (44.3)	0.57 (1)	^a^0.45
No	13 (3.4)	16 (4.1)		
Smoking for the past 1 month				
Yes	8 (2.0)	6 (1.6)	0.181 (1)	^a^0.670
No	190 (49.5)	180 (46.9)		
Alcohol for the past 1 month				
Yes	7 (1.8)	12 (3.1)	1.734 (1)	^a^0.188
No	191 (49.7)	174 (45.4)		
Caffeinated drink consumption for the past 1 month				
Yes	165 (43.0)	138 (35.9)	4.813 (1)	^a^0.028
No	33 (8.6)	48 (12.5)		

**Notes:**

a Pearson chi-square

b Fisher’s exact test

The simple logistic regression analysis identified five clinically relevant variables to be included in the MLR to determine the association between poor sleep quality and academic performance of the Malaysian university dental students. The variables were self-perceived academic performance in the current academic year, falling asleep during class, skipping class, coming late to the class, and involvement in extracurricular activities. The multiple logistic regression analysis found that two variables were significantly associated with poor sleep quality. The variables were self-perceived academic performance and skipping class (Table 6). These results can be interpreted as follows: 1) Students who self-perceived poor academic performance had higher odds of poor sleep quality by 2.95 times than students who self-perceived very good/good academic performance (Adjusted Odds Ratio (AOR) 2.95, 95% CI [1.25–6.96], *P*-value = 0.013), 2) Students who skipped class had 1.7 higher odds of poor sleep quality than students who did not skip class (AOR 1.70, 95% CI [1.00–2.91], *P*-value = 0.046). The model met the assumptions with the Hosmer–Lemeshow goodness of fit test *P*-value = 0.995, the percentage of correct classification = 55.2%, and the area under ROC curve = 0.6. There are no interaction and multicollinearity problems.

**Table 6:** Association between poor sleep quality and academic performance of Malaysian University dental students (*n* = 384).

Variable	Crude OR^a^ (95% CI)	Adjusted OR^b^ (95% CI)	Wald^b^ statistic (df)	*P*-value^b^
Self-perceived academic performance				
Very good/good	1.00	1.00		
Average	1.48 [0.91–2.40]	1.50 [0.92–2.45]	2.67 (1)	0.102
Poor	2.95 [1.25–6.96]	2.99 [1.26–7.08]	6.21 (1)	0.013
Skip class				
No	1.00	1.00		
Yes	1.70 [1.00–2.91]	1.73 [1.01–2.97]	3.98 (1)	0.046

**Notes:**

a Simple logistic regression

b Multiple logistic regression

OR = Odds ratio

CI = Confidence interval

df = degree of freedom

The Hosmer–Lemeshow goodness of fit test *P*-value = 0.995

The percentage of correct classification = 55.2%

The area under ROC curve = 0.6

There are no interaction and multicollinearity problems.

## Discussion

Studying in medical or dentistry requires a substantial commitment of time and effort from students, leading to prolonged periods of study that may adversely affect their sleep quality (BaHammam et al., 2012). The prevalence rates of poor sleep quality exhibit notable variation across various university professional courses, such as medicine, pharmacy, and dentistry, even when the same assessment tool, the PSQI, is used (Hanapi et al., 2021; Nurismadiana & Lee, 2018; Said et al., 2020). This current study used the PSQI to measure Malaysian university dental students’ sleep quality. In this study, the mean of sleep hours per night for all students was 5.72 (SD 1.06) h and 39.6% slept for 5–6 h per night. Our finding was comparable with a study conducted among dental students in Saudi Arabia (Elagra et al., 2016). A study among medical and pharmacy students in Malaysia found that 35.3% and 59% of the respondents slept about 5 to 6 h per night (Hanapi et al., 2021; Said et al., 2020). According to the American Academy of Sleep Medicine and Sleep Research Society, adults require at least 7 h of normal sleeping hour per day (Watson et al., 2015). However, children and adolescents require an average sleep time of nearly 9 h per night (Mercer, Merritt & Cowell, 1998).

The current study showed that 51.6% of students reported having poor sleep quality, which was lower than studies conducted among dental students in Saudi Arabia and Brazil where 72.5% and 65.2% of the students have poor sleep quality (Elagra et al., 2016; Muñoz et al., 2023). The findings of the present study were comparable with studies conducted among university students in China (50.2%), Taiwan (54.7%) and Hong Kong (58%) (Cheng et al., 2012; Ji & Wang, 2018; Suen, Tam & Hon, 2010). Furthermore, the study revealed a lower prevalence of poor sleep quality compared to the local studies conducted among pharmaceutical students at Universiti Sains Malaysia (84%), health sciences students at Universiti Putra Malaysia (70.6%), and medical students at International Islamic University Malaysia (59.6%) (Hanapi et al., 2021; Nurismadiana & Lee, 2018; Said et al., 2020). The disparities in the prevalence of poor sleep quality among university students may be primarily due to differences in the courses offered, sample population background, and curriculum structures across these universities. In addition, research has identified variations of sleep duration among individuals of different cultures, suggesting that sleep patterns vary substantially among different cultural populations (Cheung et al., 2021). A meta-analysis of adolescent sleep data across 23 countries from the past 30 years showed that total sleep time in Asian countries was 40–60 min shorter than in North America, and 60–120 min shorter than in Europe and Australia (Olds et al., 2010). The cultural differences in sleep practices could contribute to the different sleep duration in our study. The study findings suggest that Malaysian dental students tend to have poor sleep quality, which they may not be aware of. The results underscore the importance of addressing sleep quality among dental students, and it may be necessary to implement tailored interventions to promote adequate sleep duration and quality, which might lead to improved academic performance and overall well-being.

This study found that sleep quality was significantly associated with students’ ethnicity, those who stayed at the hostel, and those who consumed caffeinated drinks. Specifically, Malays were found to have poorer sleep quality compared to other racial groups. This finding was consistent with a study among health sciences students in Malaysia, which reported a higher percentage (52.7%) of poor sleep quality among Malay students than other races (Nurismadiana & Lee, 2018). Similarly, an earlier study conducted in Singapore reported an association between ethnicity and sleep disturbance, with Malays and Chinese having a higher prevalence of sleep disturbance than those of the Indian ethnicity (Ng & Tan, 2005).

Most of the students in this study resided in a hostel rather than outside accommodation. Notably, students’ who stayed at the hostel demonstrated significantly poor sleep quality. This finding was aligns with a study conducted among university students in Malaysia and Hongkong, which reported a high prevalence of poor sleep quality among those living in hostels (Nurismadiana & Lee, 2018; Tsui & Wing, 2009). In contrast, a separate study conducted among undergraduate health sciences students in Malaysia found that students who resided outside the campus have a five times greater risk of developing poor sleep quality compared to their on campus counterparts (Saat et al., 2020). The living environment could plays an important role in students’ sleep quality and practice. Students living on-campus tend to delay their sleep phases on weekdays and weekends, resulting in poorer sleep quality (Tsui & Wing, 2009). In addition, students may encounter stressful situations when adapting to a new environment, particularly in hostels with issues such as maintenance problems, noise, inadequate facilities, and substandard accommodation. These factors are particularly prevalent in free academic housing or hostels. On the other hand, students living outside of hostels, such as living with their parents, tend to experience better sleep quality. This could be attributed to the familiarity of their living environment, better support systems, a greater sense of security and more personal space, which can contribute to improved sleep habits (Araújo et al., 2014; Tsui & Wing, 2009).

Additionally, lifestyle factors are often associated with individual sleep quality. Findings from this study revealed that respondents who consumed caffeinated drinks were more likely to have poor sleep quality. Similar results were reported by Yilmaz, Tanrikulu & Dikmen (2017) and Lemma et al. (2012). Contrarily, a previous study by Lund et al. (2010) found caffeine consumption was not a significant predictor of sleep quality. Caffeinated drink is commonly consumed to help offset fatigue. However, it can have several adverse effects on sleep quality and quantity. Evidence suggests that higher total caffeine consumption was associated with decreased bedtime, and greater caffeine consumption was associated with reduced sleep quality (Watson et al., 2016).

Our study found no significant associations between sleep quality and age, gender, academic years, BMI, medical problems, exercise, use of electronic devices, smoking, and alcohol consumption. Gender, academic years and BMI have been identified as factors affecting the quality of an individuals’ sleep in previous studies. Females with lower BMI tend to have poor sleep quality (Cheng et al., 2012; Nurismadiana & Lee, 2018). In contrast, other study reported that higher BMI was associated with higher sleep disturbance and shorter average sleep duration scores (Narang et al., 2012). A study conducted among medical students in Malaysia found that clinical years students were less likely to have poor sleep quality compared to pre-clinical students (Said et al., 2020). Similar to our findings, Yilmaz, Tanrikulu & Dikmen (2017) reported age, gender, and academic years did not affect sleep quality. Studies have shown that electronic media use is related to sleep quality (Lavender, 2015). This aligns with a previous study conducted among Jordanian dental students which demonstrated that high smartphone addiction is significantly associated with poor sleep quality (Sanusi et al., 2022). Our study found that more than 90% of dental students used electronic devices before sleep. The previous study also found that exercise, alcohol consumption, and the presence of chronic diseases (Yilmaz, Tanrikulu & Dikmen, 2017) were not significant predictors of sleep quality.

Our result showed that students who self-perceived poor academic performance had higher odds of poor sleep quality by 2.95 times than students who self-perceived very good/good academic performance. Our finding was consistent with a study conducted among undergraduate dental students in Brazil which that found that, poor sleep quality negatively impacts the academic performance (Muñoz et al., 2023). Similar findings were reported in a study conducted among dental students in Saudi Arabia which found a significant negative correlation between sleep quality and the students’ grade point averages (GPAs). Poor sleep quality was associated with lower academic performance, especially in clinical years (Elagra et al., 2016). Dental school students have a different study rhythm in the clinical years compared to nonclinical years (BaHammam et al., 2012). The study and practice of dentistry, particularly during the clinical years, necessitate high concentration, cognitive performance, and motor dexterity. Poor sleep quality or sleep deprivation can negatively impact performance (Lemma et al., 2014). In addition, poor sleep quality and daytime lethargy reduce the levels of attention, increase anxiety, affect cognitive performance, and may impair memory and decision-making (Lo et al., 2014).

This study also showed that students who skipped class had 1.7 higher odds of poor sleep quality than students who did not skip class. Inadequate sleep quality results in fatigue, loss of concentration, a low pain threshold, anxiety, restlessness, irrational thoughts, and irritability, among other symptoms (Hayashino et al., 2010). Consequently, students with poor sleep quality are typically less motivated to attend class and tend to avoid class (Lai & Say, 2013). However, a study conducted at two tertiary institutions in Malaysia found no significant difference in the frequency of skipping class between good and poor-quality sleepers (Lai & Say, 2013).

Despite the findings, this study has several limitations. Firstly, data on academic performance and sleep quality were self-reported, which may be susceptible to recall bias. Validated and reliable questionnaires were employed to enhance data on sleep quality and minimize this bias. Secondly, the study was prone to selection bias due to the use of convenient sampling method required by time constraints. The third limitation is related to the measurement of exercise and alcohol intake, where the respondents were only asked to answer “yes” or “no” for the item. Furthermore, the questionnaire only captured the duration and frequency of exercise. It did not assess the specific type of exercise performed, such as moderate-to-vigorous or moderate activities. The main strength of our study was the participation of dental students from multiple universities across Malaysia, enhancing the generalizability of its findings. This approach facilitates a more comprehensive understanding of sleep quality issues within the diverse educational and cultural contexts encountered by dental students.

## Conclusion

About half of the dental students in Malaysia have poor sleep quality. Ethnicity, accommodation, and caffeine consumption have been identified as significant factors associated with sleep quality. Furthermore, the study also found a significant association between poor sleep quality and self-perceived poor academic performance and skip classes. The findings hihglight the importance of raising awareness to improve sleep quality among dental students, thus enabling them to better cope with the demanding learning environment of dental school.

## Supplemental Information

10.7717/peerj.17522/supp-1 Supplemental Information 1 Questionnaire-BM.

10.7717/peerj.17522/supp-2 Supplemental Information 2 STROBE.

10.7717/peerj.17522/supp-3 Supplemental Information 3 Raw Data.

## Additional Information and Declarations

### Data Availability

The following information was supplied regarding data availability: The raw data is available in the Supplemental File.
