# Supplementary material for: Factors associated with poor sleep quality among dental students in Malaysia
Source: PeerJ. 2024 Jun 27;12:e17522. doi: 10.7717/peerj.17522 (PMC11214741; doi:10.7717/peerj.17522)
Supplement: Supplemental Information 1 [file peerj-12-17522-s001.docx]

**Appendix A: Borang Soal Selidik**

**Tajuk: Soal Selidik Berkenaan Faktor-Faktor Berkaitan Kualiti Tidur Yang Lemah dalam Kalangan Pelajar Pergigian di Malaysia**

**Bahagian A: Maklumat Peribadi Pesakit**

**Maklumat sosiodemografi**

Sila tandakan (√) pada jawapan yang sesuai

| 1 | Umur | ___________ tahun | | | |
| --- | --- | --- | --- | --- | --- |
| 2 | Tahun akademik semasa | ☐Tahun 2  ☐Tahun 3  ☐Tahun 4  ☐Tahun 5 | | | |
| 3 | Jantina | □ Lelaki | □ Perempuan | | |
| 4 | Kumpulan etnik | □ Melayu | □ Cina | □ India | □ Lain-lain, Nyatakan ___________ |
| 5 | Pendapatan Bulanan Keluarga | RM _____________________ (Nyatakan) | | | |
| 6 | Taraf perkahwinan | □ Bujang | □ Berkahwin | □ Bercerai | □ Balu |
| 7 | Jisim Indek Badan | Berat:____________(kg)  Tinggi:___________(cm) | | | |
| 8 | Tempat Belajar | ☐Universiti Sains Malaysia (USM)  ☐Universiti Malaya (UM)  ☐International Islamic University Malaysia (IIUM)  ☐Universiti Teknologi MARA (UiTM) | | | |
| 9 | Dimana anda tinggal | ☐Asrama  ☐Rumah sewa/ Rumah keluarga | | | |
| 10 | Adakah anda mempunyai sebarang masalah perubatan? | ☐Ya ☐Tidak  If ya, nyatakan masalah perubatan anda:_________________________ | | | |

**BAHAGIAN B: Aktiviti atau Tabiat Berkaitan Kualiti Tidur**

**Sila ingat aktiviti atau tabiat anda yang boleh menjejaskan tidur anda sejak SEBULAN LEPAS.**

**(Sila tandakan ∕ pada petak yang disediakan)**

| **1.** | Adakah anda bersenam dalam masa sebulan yang lepas?  (cth: Berjoging, gimnasium, mendaki, berenang) | ☐Ya ☐Tidak  *Jika tidak, sila teruskan ke soalan 3* |
| --- | --- | --- |
| **2.** | Kekerapan senaman selama sebulan yang lalu | >5 kali seminggu  3-5 kali seminggu  <3 kali seminggu  ******* ***Tempoh setiap senaman hendaklah sekurang-kurangnya 30 minit setiap sesi*** |
| **3.** | Adakah anda mengambil sebarang pil tidur untuk memperbaiki quality tidur selama sebulan yang lalu? | ☐Ya ☐Tidak |
| **4.** | Adakah anda menggunakan peranti elektronik sebelum tidur selama sebulan yang lalu?  *(cth: handphone, laptop, tablet)* | ☐Ya ☐Tidak  *Jika tidak, teruskan ke soalan 6* |
| **5.** | Kekerapan menggunakan peranti elektronik sebelum tidur selama sebulan yang lalu | >5 kali seminggu  3-5 kali seminggu  <3 kali seminggu |
| **6.** | Adakah anda merokok sejak sebulan yang lalu? | ☐Ya ☐Tidak  *Jika anda seorang perokok, sila nyatakan berapa batang rokok sehari:_________________________* |
| **7.** | Adakah anda pernah minum alkohol selama sebulan yang lalu? | ☐Ya ☐Tidak |
| **8.** | Adakah anda mengambil minuman berkafein sejak sebulan yang lalu**?**  *(*Cth*: Kopi, teh, nescafe )* | ☐Ya ☐Tidak  *Jika tidak, teruskan ke bahagian C.* |
| **9.** | Kekerapan minum minuman berkafein sejak sebulan yang lalu  (Cth: Kopi, teh, nescafe ) | Jarang mengambil minuman berkafein  1 hari seminggu  2-3 hari seminggu  4-6 hari seminggu  Setiap hari |
| **10.** | Sila nyatakan purata cawan minuman berkafein yang diambil setiap minggu.  *(cth: 1cawan sehari x 7hari= 7cawan seminggu)* |  |

**BAHAGIAN C: PRESTASI AKADEMIK**

**Sila jawab soalan untuk SEBULAN LEPAS.**

**(Sila tandakan ∕ pada petak yang disediakan)**

| **1.** | **Bagaimanakah anda melihat prestasi akademik keseluruhan anda selama sebulan yang lalu?** | ☐ Sangat bagus  ☐ Bagus  ☐ Sederhana  ☐ Tidak bagus  ☐ Sangat tidak bagus |
| --- | --- | --- |
| **2.** | **Sila nyatakan purata mata gred kumulatif (CGPA) anda untuk tahun akademik yang lalu?** | ___________( e.g. : 0.00 to 4.00)  *Jika tiada CGPA, sila tandakan 0.* |
| **3.** | **Adakah anda tertidur semasa kelas selama sebulan yang lalu?** | ☐Tidak pernah  Jarang  Kadang-kadang  Selalu  Sangat selalu |
| **4.** | **Adakah anda pernah ponteng kelas selama sebulan yang lalu?** | ☐Ya ☐Tidak |
| **5.** | **Jika ya , berapa kali anda ponteng kelas selama sebulan yang lalu?** | ☐<3 kali sebulan kerana sakit  ☐3 kali atau lebih sebulan kerana sakit  ☐<3 kali sebulan atas sebab lain  ☐3 kali atau lebih sebulan atas sebab lain |
| **5.** | **Adakah anda datang lewat ke kelas sejak sebulan yang lalu?** | ☐Ya ☐Tidak |
| **6.** | **Jika ya , berapa kali anda datang lewat ke kelas selama sebulan yang lalu?** | ☐< 3 kali seminggu kerana bangun lewat  ☐3 kali seminggu kerana bangun lewat  ☐<3 kali seminggu atas sebab lain  ☐3 kali seminggu atas sebab lain |
| **7.** | **Do you involve in any extracurricular activities for the past one month?** | ☐Tidak pernah  Jarang  Kadang-kadang  Selalu  Sangat selalu |

**BAHAGIAN D: SOAL SELIDIK INDEKS KUALITI TIDUR PITTBURGH (PSQI-M)**

ARAHAN: Soalan-soalan berikut adalah mengenai tabiat tidur anda pada kebiasaannya dalam tempoh sebulan (30 hari) yang lalu. Jawapan anda harus menggambarkan keadaan yang paling tepat bagi kebanyakan waktu siang dan malam anda dalam sebulan yang lalu. Sila jawab semua soalan.

| 1. | Pada pukul berapa biasanya anda masuk tidur? | | | | | ______________ | | | | |
| --- | --- | --- | --- | --- | --- | --- | --- | --- | --- | --- |
| 2. | Berapa lamakah masa diambil untuk anda lelap pada setiap malam? | | | | | ______________ minit | | | | |
| 3. | Pukul berapakah kebiasaannya anda bangun di waktu pagi? | | | | | ______________ | | | | |
| 4. | Berapa jumlah jam tidur sebenar yang anda biasa perolehi pada waktu malam?  (Ini mungkin berbeza dengan jumlah jam yang anda luangkan di atas katil) | | | | | ______________ jam | | | | |
| 5. Dalam sebulan yang lalu, berapa kerapkah anda mengalami masalah tidur kerana anda … | | | | | | | | | | |
|  | | Tidak pernah dalam sebulan lalu | | Kurang daripada sekali seminggu | | | Satu atau dua kali seminggu | | Tiga kali atau lebih seminggu | |
| a) Tidak boleh lelap dalam tempoh masa 30 minit | |  | |  | | |  | |  | |
| b) Terjaga di tengah malam atau awal pagi | |  | |  | | |  | |  | |
| c)Perlu bangun tidur untuk ke tandas | |  | |  | | |  | |  | |
| d) Tidak boleh bernafas dengan selesa | |  | |  | | |  | |  | |
| e) Batuk atau berdengkur dengan kuat | |  | |  | | |  | |  | |
| f) Berasa sangat sejuk | |  | |  | | |  | |  | |
| g) Berasa sangat panas | |  | |  | | |  | |  | |
| h) Mengalami mimpi buruk | |  | |  | | |  | |  | |
| i)Mengalami kesakitan | |  | |  | | |  | |  | |
| j) Sila nyatakan sebab lain dan berapa kerap anda mengalami masalah tidur disebabkan ini | | Sebab lain ______________________________  Kekerapan ______________________________ | | | | | | | | |
| 6. | Dalam sebulan yang lalu, berapa kerapkah anda mengambil ubat (ubat yang dinasihati oleh doktor atau ubat yang dibeli sendiri tanpa preskripsi untuk membantu anda tidur?) | |  | |  | | |  | |  |
| 7. | Dalam sebulan yang lalu, berapa kerapkah anda mengalami masalah untuk berjaga semasa memandu kenderaan, makan, atau Ketika melibatkan diri dengan aktiviti sosial? | |  | |  | | |  | |  |
| 8. | Dalam sebulan yang lalu, adakah menjadi suatu masalah untuk anda kekal bersemangat dalam menyelesaikan kerja? | | ☐Tiada masalah langsung  ☐Sedikit masalah  ☐Agak bermasalah  ☐Masalah yang besar | | | | | | | |
| 9. | Dalam sebulan yang lalu, bagaimanakah anda nilai kualiti tidur anda secara keseluruhan? | | ☐Sangat baik  ☐Agak baik  ☐Agak teruk  ☐Sangat teruk | | | | | | | |
